# Bedside ultrasound-assisted manual reduction in ultrasound-selected, non-transient pediatric intussusception: a single-center experience

**DOI:** 10.3389/fmed.2026.1787917

**Published:** 2026-04-29

**Authors:** Xing Zhang, HongYan Chen, Jing Xiong

**Affiliations:** 1 Department of Clinical Laboratory, Wuhan Fourth Hospital, Wuhan, China; 2 Department of Medical Ultrasound, Wuhan Fourth Hospital, Wuhan, China

**Keywords:** clinical efficacy, intussusception, manual reduction, pediatrics, ultrasound guidance

## Abstract

**Purpose:**

To evaluate the feasibility and safety of bedside, single-operator ultrasound-assisted manual reduction (UAMR) in ultrasound-selected, non-transient pediatric intussusception in routine clinical practice.

**Methods:**

We retrospectively reviewed 119 ultrasound-selected, non-transient pediatric intussusception encounters managed with bedside UAMR between March 2023 and December 2025. Encounter-level baseline characteristics and index-encounter UAMR outcomes were summarized. Because standardized segment-length measurements and post-discharge recurrence data were not uniformly available in the medical records, the analysis focused on documented clinical characteristics, repeat-attempt patterns, overall bedside success, and the absence of major documented complications.

**Results:**

Among 119 encounters, the median age was 5.0 years (IQR 3.0–6.0), and 58 (48.7%) were boys. Multiple intussusceptions, lymph node enlargement, and ascites were documented in 12 (10.1%), 60 (50.4%), and 10 (8.4%) encounters, respectively. UAMR achieved complete resolution on the first attempt in 111/119 encounters (93.3%); 6 additional encounters resolved after repeated bedside attempts (5 on the second attempt and 1 on the third), yielding an overall index-encounter success rate of 117/119 (98.3%). Two encounters remained unreduced. No perforation or bleeding was documented in the available records. The recorded check-to-report interval had a median of 13.6 min (IQR 9.1–29.2).

**Conclusion:**

In this single-center series, bedside UAMR showed high index-encounter success and no documented major procedural complications among ultrasound-selected, non-transient pediatric intussusception encounters. Prospective studies with standardized eligibility criteria, stopwatch-measured manipulation time, and structured recurrence follow-up are needed before broader practice recommendations can be made.

## 1 Introduction

Ileocolic intussusception is the most common abdominal emergency in infants and toddlers, with the highest incidence in the first 3 years of life (1, 2). By contrast, ultrasound also identifies a group of small-bowel lesions that are shorter, non-fixed, and often self-limited. Sonography therefore not only confirms intussusception, but also provides subtype cues: non-fixed lesions away from the right lower quadrant, short segment length, preserved mural perfusion, and the absence of a lead point are more compatible with small-bowel intussusception, whereas a fixed right lower quadrant/ileocecal mass with greater extent more strongly suggests ileocolic disease (3, 4).

Conventional non-surgical treatment mainly relies on pneumatic or hydrostatic enema reduction under fluoroscopic or ultrasound guidance. These methods remain effective and widely accepted (4–6), but they usually require additional personnel, dedicated radiologic resources, and, in some cases, sedation or analgesia (7–10). Moreover, because many small-bowel lesions may resolve spontaneously, routine enema is not appropriate for all ultrasound-detected intussusceptions. The practical problem in daily care is the persistently symptomatic, non-transient child whose lesion remains visible on repeat ultrasound and who continues to have colicky pain, vomiting, or recurrent emergency presentations despite short-interval observation.

Ultrasound-assisted manual reduction (UAMR) is a newer bedside strategy intended to address some of these limitations (11, 12). Prior reports from specialized centers have described ultrasound-assisted manual reduction of ileocolic intussusception with encouraging outcomes; in one series, manual reduction alone achieved complete reduction in 80% of pediatric cases, with an overall 93% non-surgical success rate when combined with subsequent enema when needed (12). Rather than advocating intervention for all SBI, we hypothesized that a standardized bedside UAMR workflow could be safely applied in carefully selected children with ultrasound-selected, non-transient pediatric intussusception-most commonly SBI-leaning lesions-thereby potentially expediting symptom relief while avoiding radiation and routine sedation. Accordingly, we retrospectively analyzed 119 consecutive encounters managed with a simple bedside, single-operator UAMR protocol. UAMR was reserved for hemodynamically stable children with ongoing symptoms and lesions persisting on repeat ultrasound after a 45–60-min observation/reassessment interval under predefined safety criteria, while incidentally detected transient lesions that resolved spontaneously were managed conservatively and not included. Our aim was to describe the feasibility and apparent safety of UAMR in ultrasound-selected, non-transient pediatric intussusception and to explore how this bedside strategy may fit into real-world care after short-interval sonographic reassessment.

## 2 Materials and methods

### 2.1 Study design

We conducted a retrospective observational study at Wuhan Fourth Hospital, an urban tertiary hospital in Wuhan, China, operating across three campuses (Wusheng, Gutian, and Changqing) with more than 2,600 beds. The pediatric service receives approximately 55,000 visits annually, with marked seasonal fluctuation. The study included 119 ultrasound-selected, non-transient pediatric intussusception encounters managed with bedside UAMR between March 2023 and December 2025. Diagnosis was based on compatible clinical presentation and characteristic ultrasonographic findings, including the transverse target/doughnut sign and the longitudinal pseudokidney sign. The study was approved by the Ethics Committee of Wuhan Fourth Hospital (KY2026-044-01).

### 2.2 Inclusion/exclusion criteria

Pediatric patients were eligible if they had sonographically confirmed intussusception and underwent bedside UAMR under our institutional workflow. UAMR was offered only to hemodynamically stable children whose symptoms and repeat ultrasound remained compatible with persistent intussusception after a 45–60-min observation/reassessment interval and whose sonographic findings suggested a potentially reducible, non-ischemic lesion (13, 14). Children with rapid symptom resolution or disappearance of sonographic findings during this interval, including transient-appearing small-bowel intussusceptions managed conservatively, did not proceed to bedside UAMR and were therefore not included in the present dataset.

Exclusion criteria comprised clinical or ultrasonographic signs of bowel ischemia, perforation, generalized peritonitis, or advanced obstruction; definite or strongly suspected pathological lead points (e.g., intestinal polyps, Meckel’s diverticulum, neoplasms, duplication cysts); features suggestive of chronic or recurrent intussusception warranting direct surgical assessment; hemodynamic instability or severe systemic illness such as septic shock; and cases primarily allocated to standard enema-first or surgery-first pathways according to institutional practice. Patients were also excluded if informed consent from legal guardians could not be obtained.

### 2.3 Ultrasound assessment

All patients underwent abdominal ultrasound examination using a Mindray Resona A20T ultrasound platform (Shenzhen Mindray Bio-Medical Electronics Co., Ltd., Shenzhen, China) with a 3.5–5.0 MHz convex transducer and color Doppler capability. The diagnosis of intussusception was based on classic target and pseudokidney appearances, together with real-time assessment of bowel wall perfusion and proximal bowel dilatation when present. The same ultrasound platform was used during the bedside maneuver to provide real-time guidance and immediate confirmation of reduction or persistence.

### 2.4 Operator training and experience

All UAMR procedures were performed independently by a single attending physician experienced in pediatric ultrasonography and bedside reduction techniques. Before independent practice, the operator completed 2 months of structured in-department training and 10 supervised UAMR procedures, thereby reducing inter-operator variability in this single-center series.

### 2.5 Description of ultrasound-assisted manual reduction technique

**Figure 1:**
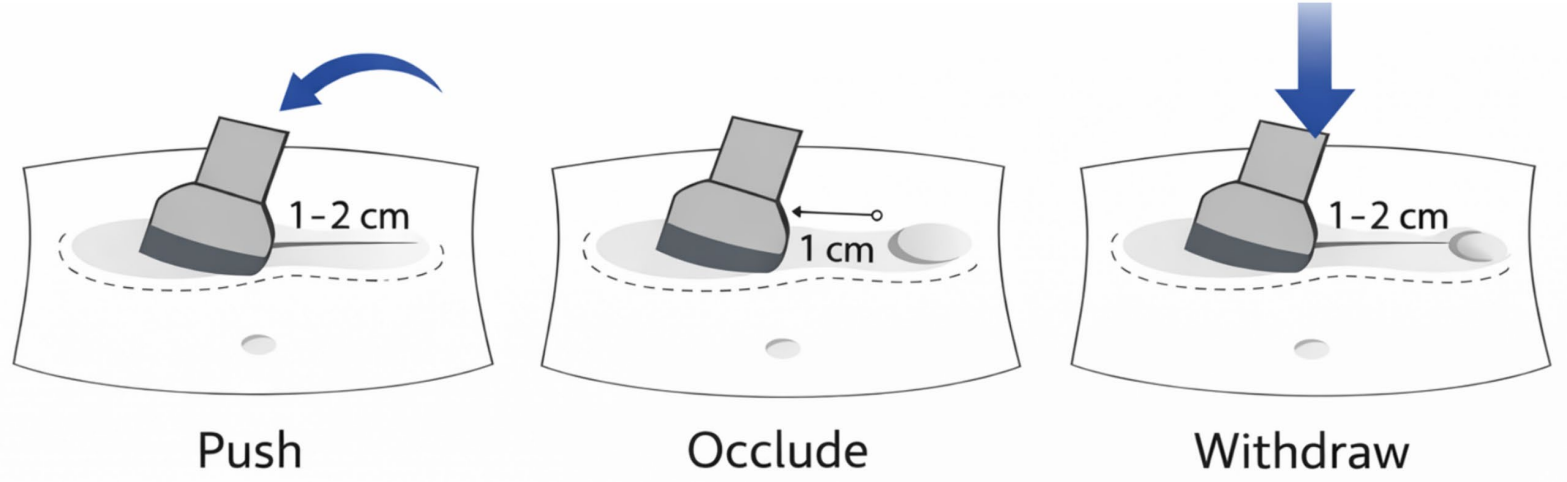
Schematic illustration of the standardized bedside UAMR maneuver using a stepwise push–occlude–withdraw strategy. Push: the probe gently nudges the tip of the intussusceptum along the bowel axis to shorten the invaginated segment. Occlude: the probe is repositioned approximately 1 cm distal on the intussuscipiens to transiently appose the bowel walls and create a controlled distal occlusion. Withdraw: light pressure is maintained through several peristaltic cycles to favor retrograde withdrawal of the intussusceptum, with stepwise repositioning of the occlusion point as reduction advances. Compression was intentionally gentle, typically corresponding to approximately 1–2 cm of abdominal wall depression, and each compression cycle was maintained for about 10–60 s.

A single operator performed UAMR at the bedside without enema and without routine sedation, under continuous real-time ultrasound guidance. A curvilinear 3–5 MHz transducer provided panoramic monitoring, and the probe itself served as the manipulating instrument. Sedation was not part of the routine local workflow because the maneuver relied on gentle graded compression with continuous sonographic feedback, and contemporary pediatric intussusception practice shows marked international variation in analgesia/sedation use, with many centers still performing nonoperative reduction in awake children (7–10) (see Figure 1).

**Figure 2:**
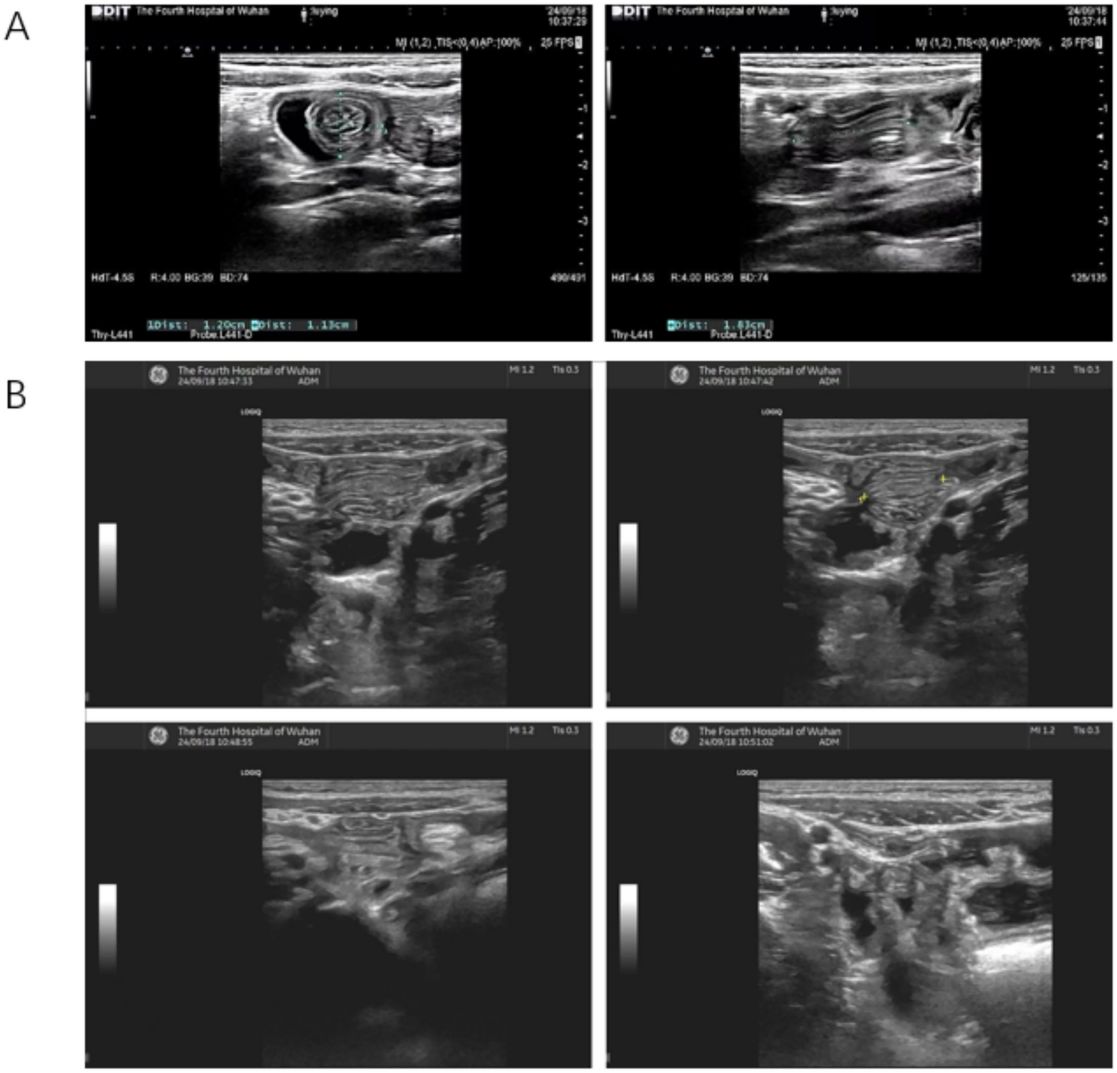
Representative real-time ultrasonographic images illustrating the diagnosis and successful manual reduction of intussusception. **(A)** Initial ultrasound image obtained at 10:37 a.m., demonstrating the classic “target sign” suggestive of intussusception. **(B)** Sequential ultrasound snapshots taken between 10:47 and 10:51 a.m. during the manual reduction procedure. The length of the intussuscepted segment progressively decreased, culminating in complete reduction and resolution of the “target sign”.

The operator identified the intussusception, delineated the intussusceptum and intussuscipiens, and mapped peristaltic direction. Using graded tangential probe pressure, the operator (i) gently nudged the tip of the intussusceptum along the bowel axis to shorten the invaginated segment (push), (ii) positioned the probe approximately 1 cm distal on the intussuscipiens to transiently appose its walls and create a controlled distal occlusion (occlude), and (iii) maintained light pressure through several peristaltic cycles to favor retrograde withdrawal of the intussusceptum, repositioning the occlusion point stepwise as reduction advanced (withdraw). Compression was intentionally gentle, typically corresponding to approximately 1–2 cm of abdominal wall depression, and each compression cycle was maintained for about 10–60 s. In routine charting, exact pressure values and formal cycle counts were not recorded as structured variables; therefore, the maneuver is described here using pragmatic semiquantitative language that reflects actual bedside practice. The maneuver was terminated immediately if the child developed escalating pain, worsening tenderness or guarding, ultrasonographic evidence of deteriorating mural perfusion or progressive bowel edema, increasing proximal bowel dilatation/obstruction, or any clinical concern for perforation or peritoneal irritation. In such situations, the child was escalated for immediate surgical assessment and operative management if required. Representative real-time ultrasonographic images of diagnosis and bedside reduction are shown in Figure 2.

### 2.6 Outcome measures

The primary outcome was index-encounter bedside UAMR success, defined as documented complete resolution during the encounter. Secondary outcomes included first-attempt success, the need for repeat bedside attempts during the same encounter, and documented major adverse events such as perforation or bleeding. Post-discharge recurrence was not analyzed as a primary endpoint because it was not uniformly captured in the medical records.

The medical records contained timestamps for ultrasound check time and report time. The difference between these timestamps was summarized as the recorded check-to-report interval. This variable was treated as a workflow surrogate rather than a stopwatch-measured manipulation duration, because documentation delay and reporting workflow could contribute to longer intervals in some encounters.

### 2.7 Follow-up and monitoring

Following bedside reduction, children were observed clinically in the pediatric observation unit according to routine practice. A repeat color Doppler ultrasound was usually obtained approximately 45 min later. This short-interval reassessment strategy was intended to verify persistent reduction while allowing transient bowel edema or spontaneously resolving episodes to declare themselves, and it is consistent with published repeat-ultrasound approaches using reassessment at 45 min and, when needed, at 45-min intervals (14, 15). If no residual intussusception was detected and the child remained clinically stable, discharge with home observation was recommended (16–19). Caregivers were advised to monitor for recurrent abdominal pain, vomiting, irritability, or other concerning symptoms and to return promptly if these occurred. A routine telephone follow-up was conducted 2 days after discharge. If the initial reduction was incomplete, repeat bedside UAMR could be considered after reassessment, but no more than three bedside attempts were performed during a single encounter. Persistent failure after repeated attempts, or the emergence of concerning clinical or ultrasonographic findings, prompted escalation to surgical consultation or referral as appropriate.

### 2.8 Data analysis

Given the retrospective observational design, the analysis was primarily descriptive. Continuous variables were summarized as medians with interquartile ranges and ranges, and categorical variables as frequencies and percentages. Presenting symptoms (vomiting, abdominal pain, diarrhea, fever, and reduced activity/poor spirit) were abstracted from the free-text history field of the medical records and coded descriptively when explicitly documented. Comorbidity data were not uniformly preserved as structured variables and therefore were not tabulated separately. Spearman’s rank correlation was used to explore the association between patient age and the recorded check-to-report interval. Because no between-group comparative analysis was prespecified, *p*-values were not added to the descriptive tables. Statistical analyses were performed using SPSS Statistics (IBM Corp., Armonk, NY, United States). A two-sided *p*-value <0.05 was considered statistically significant.

## 3 Results

A total of 119 ultrasound-selected, non-transient pediatric intussusception encounters managed with bedside UAMR were included in the analysis. The cohort included 58 boys (48.7%) and 61 girls (51.3%), with an age range from 7 months to 14 years and a median age of 5.0 years (IQR 3.0–6.0 years). Most encounters were outpatient presentations (117/119, 98.3%). Multiple intussusceptions were documented in 12 encounters (10.1%), lymph node enlargement in 60 (50.4%), and ascites in 10 (8.4%) (Table 1). Presenting symptoms abstracted from the history field most commonly included vomiting (83/119, 69.7%) and abdominal pain (77/119, 64.7%), followed by reduced activity/poor spirit (24/119, 20.2%), fever (18/119, 15.1%), and diarrhea (14/119, 11.8%) (Table 2). Structured comorbidity coding was not consistently available across encounters and was therefore not summarized separately.

**Table 1:** Baseline characteristics and bedside UAMR outcomes (*N* = 119).

Variable	Overall
Age, years, median (IQR)	5.00 (3.00–6.00)
Age range, years	0.58–14.00
Male, *n* (%)	58 (48.7%)
Outpatient encounters, *n* (%)	117 (98.3%)
Multiple intussusceptions, *n* (%)	12 (10.1%)
Lymph node enlargement, *n* (%)	60 (50.4%)
Ascites, *n* (%)	10 (8.4%)
First-attempt complete resolution, *n* (%)	111 (93.3%)
Needed >1 bedside attempt, *n* (%)	8 (6.7%)
Overall index-encounter UAMR success, *n* (%)	117 (98.3%)
Documented unsuccessful UAMR, *n* (%)	2 (1.7%)
Recorded check-to-report interval, min, median (IQR)	13.6 (9.1–29.2)
Recorded interval range, min	0.8–1352.8
Documented perforation/bleeding, *n* (%)	0

**Table 2:** Presenting symptoms abstracted from free-text history (non-mutually exclusive, *N* = 119).

Symptom	Overall
Vomiting, *n* (%)	83 (69.7%)
Abdominal pain, *n* (%)	77 (64.7%)
Reduced activity/poor spirit, *n* (%)	24 (20.2%)
Fever, *n* (%)	18 (15.1%)
Diarrhea, *n* (%)	14 (11.8%)

UAMR, ultrasound-assisted manual reduction.

**Figure 3:**
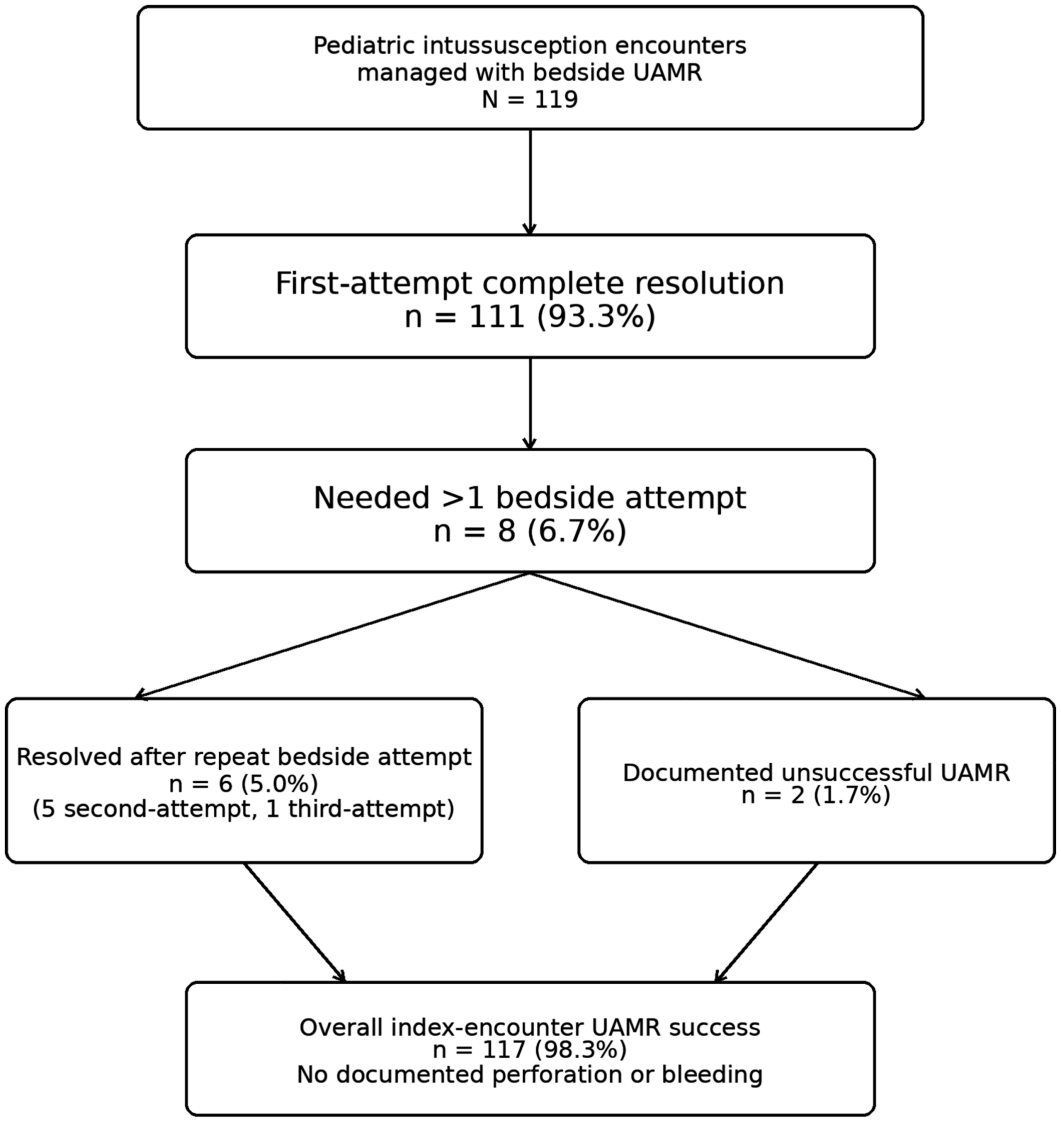
Outcome flow of the bedside UAMR cohort (*N* = 119), showing first-attempt success, repeat attempts during the same encounter, and unsuccessful cases.

Bedside UAMR achieved complete resolution on the first attempt in 111 of 119 encounters (93.3%). Eight encounters required more than one bedside attempt; among these, 5 were successfully reduced on the second attempt and 1 on the third attempt, whereas 2 remained unsuccessful. Accordingly, the overall index-encounter bedside success rate was 117/119 (98.3%) (Figure 3). No perforation or bleeding was documented in the available records. The recorded check-to-report interval had a median of 13.6 min (IQR 9.1–29.2 min). Spearman analysis showed no significant correlation between patient age and the recorded interval (*ρ* = 0.011, *p* = 0.909).

## 4 Discussion

This study describes a bedside, single-operator UAMR workflow applied to 119 ultrasound-selected, non-transient pediatric intussusception encounters. That restriction is important: our cohort was not intended to represent all ultrasound-detected intussusceptions, because transient or incidentally detected lesions that resolved during observation were managed conservatively and excluded. Within this selected population, first-attempt complete resolution was achieved in 93.3% of encounters, and the overall index-encounter bedside success rate reached 98.3% after limited repeat attempts, with no documented major procedural complications. Continuous ultrasound guidance permitted real-time assessment of lesion behavior, mural perfusion, and progressive reduction, while the bedside format allowed diagnosis, selection, and treatment to occur in one session.

Most prior pediatric literature centers on enema-based reduction, which remains the standard first-line treatment for uncomplicated childhood intussusception (3, 4, 6). Our experience does not challenge that standard. Rather, it suggests that a minimalistic bedside ultrasound-assisted strategy may be feasible in a narrower group of ultrasound-selected, non-transient cases, particularly when lesions appear SBI-leaning and remain symptomatic after short-interval reassessment. This distinction matters because many SBI episodes are inherently self-limiting (13, 14) and should not be interpreted as routine candidates for manipulation. For that reason, we deliberately limited UAMR to hemodynamically stable children whose intussusception persisted on repeat ultrasound after 45–60 min, and our findings should not be extrapolated to all SBI or all pediatric intussusception.

From a practical standpoint, bedside UAMR offers several potential advantages. The maneuver is radiation-free, can be performed with portable ultrasound, and in our workflow was usually undertaken without routine sedation, which may reduce resource demands and procedural burden in selected children (7–10). These features may be particularly relevant in after-hours care or settings where immediate access to radiologic enema is limited. At the same time, because spontaneous reduction remains a plausible explanation for some rapidly resolving lesions, especially among SBI-leaning cases, the observed success rate should be interpreted cautiously. Repeat ultrasound selection after the observation/reassessment interval was intended to reduce—but could not eliminate—this source of bias (13, 14).

Several limitations should be acknowledged. First, this was a retrospective single-center study performed by a single experienced operator, which limits generalizability. Second, structured documentation of segment length, formal subtype labels, symptom duration, exact force or cycle counts, and recurrence beyond routine 2-day telephone follow-up was incomplete in some encounters, which limited more granular subgroup analyses. Third, there was no contemporaneous observation-only, enema-first, or surgery-first comparison group. Finally, because subtype assignment was sonographic rather than surgical or pathologic, some degree of selection or classification error remains possible. Nonetheless, our findings suggest that bedside UAMR may serve as a radiation-free adjunctive option for carefully selected children with ultrasound-selected, non-transient pediatric intussusception within experienced ultrasound workflows. In our view, its current value lies less in replacing standard enema than in providing a hypothesis-generating, workflow-oriented strategy for selected persistently symptomatic lesions-predominantly SBI-leaning lesions-in centers able to monitor perfusion and lesion behavior continuously at the bedside. Prospective multicenter work with standardized eligibility criteria, stopwatch-measured manipulation time, predefined follow-up, and parallel comparison arms will be needed to define the proper role and boundaries of UAMR.

## Data Availability

The raw data supporting the conclusions of this article will be made available by the authors, without undue reservation.

## References

[1] Li Y Zhou Q Liu C Sun C Sun H Li X . Epidemiology, clinical characteristics, and treatment of children with acute intussusception: a case series. BMC Pediatr. (2023) 23:143. doi: 10.1186/s12887-023-03961-y, 36997992 PMC10061978

[2] Cox S Withers A Arnold M Chitnis M de Vos C Kirsten M . Clinical presentation and management of childhood intussusception in South Africa. Pediatr Surg Int. (2021) 37:1361–70. doi: 10.1007/s00383-021-04946-7, 34213589 PMC8408053

[3] Plut D Phillips GS Johnston PR Lee EY. Practical imaging strategies for intussusception in children. AJR Am J Roentgenol. (2020) 215:1449–63. doi: 10.2214/AJR.19.22445, 33084362

[4] Hwang J Yoon HM Kim PH Jung AY Lee JS Cho YA. Current diagnosis and image-guided reduction for intussusception in children. Clin Exp Pediatr. (2023) 66:12–21. doi: 10.3345/cep.2021.01816, 35798026 PMC9815940

[5] Caruso AM Pane A Scanu A Muscas A Garau R Caddeo F . Intussusception in children: not only surgical treatment. J Pediatr Neonat Individ Med. (2017) 6:e060135. doi: 10.7363/060135

[6] Kelley-Quon LI Arthur LG Williams RF Goldin AB St. Peter SD Beres AL . Management of intussusception in children: a systematic review. J Pediatr Surg. (2021) 56:587–96. doi: 10.1016/j.jpedsurg.2020.09.055, 33158508 PMC7920908

[7] Poonai N Cohen DM MacDowell D Mistry RD Mintegi S Craig S . Sedation and analgesia for reduction of pediatric ileocolic intussusception. JAMA Netw Open. (2023) 6:e2317200. doi: 10.1001/jamanetworkopen.2023.17200, 37285152 PMC10248743

[8] Hailemariam T Sisay S Mebratu Y Belay F Getinet T Solomon S . Effects of sedatives on radiologic enema reduction in children with ileocolic intussusception: a systematic review and meta-analysis. Eur J Radiol. (2024) 170:111237. doi: 10.1016/j.ejrad.2023.111237, 38039783

[9] Yeoh K Palmer GM Teague WJ Shavit I Babl FE. Periprocedural analgesia and sedation in air enema reduction for intussusception: a retrospective Australian cohort study. J Paediatr Child Health. (2021) 57:103–8. doi: 10.1111/jpc.15142 32902064

[10] Gal M Gamsu S Jacob R Cohen DM Shavit I. Reduction of ileocolic intussusception under sedation or anaesthesia: a systematic review of complications. Arch Dis Child. (2022) 107:335–40. doi: 10.1136/archdischild-2021-322706, 34417187

[11] Zhong Q Zhang Y You X. Ultrasound-guided manual reduction of small-intestine intussusception: a case report. Asian J Surg. (2022) 45:2105–6. doi: 10.1016/j.asjsur.2022.04.119, 35577647

[12] Vazquez JL Ortiz M Doniz MC Montero M Del Campo VM. External manual reduction of paediatric idiopathic ileocolic intussusception with US assistance: a new, standardised, effective and safe manoeuvre. Pediatr Radiol. (2012) 42:1197–204. doi: 10.1007/s00247-012-2424-0 22875204

[13] Wang G Li Z Duan G Sui B Jin Z Chu Z . Nomogram for spontaneous reduction in pediatric intussusception: a retrospective study. Front Pediatr. (2025) 13:1571203. doi: 10.3389/fped.2025.1571203, 40703312 PMC12283999

[14] Wang S Wang Y Jia L Wang X. Transient and persistent small-bowel intussusception in children: a decision tree analysis model based on ultrasound and clinical findings. BMC Gastroenterol. (2025) 25:294. doi: 10.1186/s12876-025-03839-6, 40275162 PMC12023406

[15] Qiang H Fu M Feng S Yang Y Zhu D. Repeated ultrasound-guided hydrostatic reduction for pediatric intussusception: attempt number, interval, and success. Eur J Med Res. (2025) 30:1278. doi: 10.1186/s40001-025-03562-8, 41291924 PMC12751891

[16] Whitehouse JS Gourlay DM Winthrop AL Cassidy LD Arca MJ. Is it safe to discharge intussusception patients after successful hydrostatic reduction? J Pediatr Surg. (2010) 45:1182–6. doi: 10.1016/j.jpedsurg.2010.02.085, 20620317

[17] Kwon H Lee JH Jeong JH Yang HR Kwak YH Kim DK . A practice guideline for postreduction management of intussusception of children in the emergency department. Pediatr Emerg Care. (2019) 35:533–8. doi: 10.1097/PEC.0000000000001056 28146013

[18] Arshad SA Hebballi NB Hegde BN Avritscher EBC John SD Lapus RM . Early discharge after nonoperative management of intussusception is both safe and cost-effective. J Pediatr Surg. (2022) 57:147–52. doi: 10.1016/j.jpedsurg.2021.09.047, 34756701

[19] Elliott BM Wells JM Naera S Weston A Copland J Gosavi S . Post-reduction observation and recurrence of pediatric intussusception in New Zealand: a national multicenter retrospective study. J Pediatr Surg Open. (2024) 7:100155. doi: 10.1016/j.yjpso.2024.100155

